# Supplementary material for: Association between oral microbiome and seven types of cancers in East Asian population: a two-sample Mendelian randomization analysis
Source: Front Mol Biosci. 2023 Nov 21;10:1327893. doi: 10.3389/fmolb.2023.1327893 (PMC10702768; doi:10.3389/fmolb.2023.1327893)
Supplement: Supplementary file 1 [file DataSheet1.ZIP › Supplementary Materials/MR plots of saliva/saliva═╝/Lung Cancer/pheno.2647_to_lung cancer_leave_one_out.pdf]

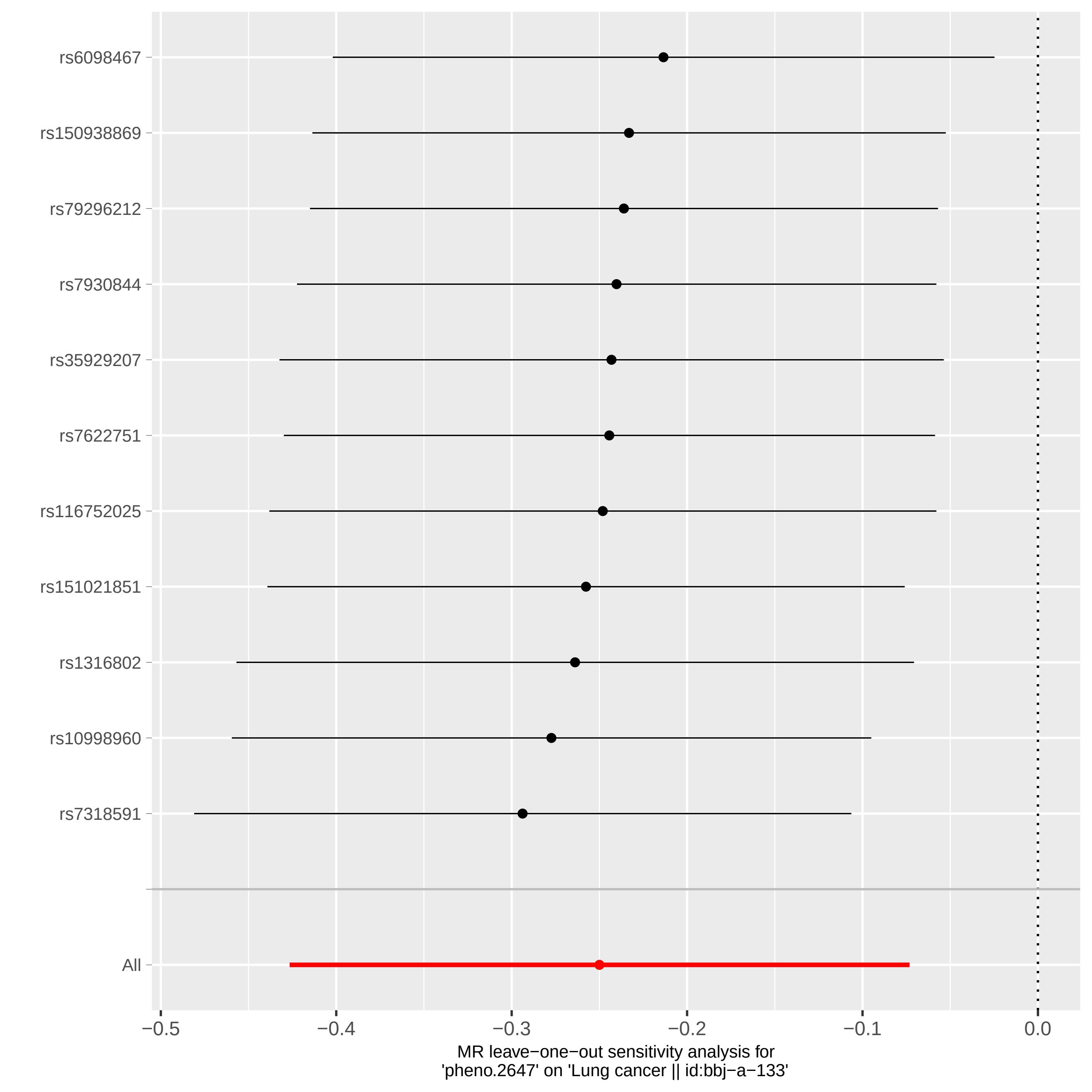
rs6098467
rs150938869
rs79296212
rs7930844
rs35929207
rs7622751
rs116752025
rs151021851
rs1316802
rs10998960
rs7318591
All
−0.5
−0.4
−0.3
−0.2
−0.1
0.0
MR leave−one−out sensitivity analysis for
'pheno.2647' on 'Lung cancer || id:bbj−a−133'
